# Natural radionuclides and radiological risk assessment of granite mining field in Asa, North-central Nigeria

**DOI:** 10.1016/j.mex.2019.10.032

**Published:** 2019-11-02

**Authors:** Muyiwa Michael Orosun, Mojisola Rachael Usikalu, Kayode John Oyewumi, Theophilus Aanuoluwa Adagunodo

**Affiliations:** a Department of Physics, University of Ilorin, Ilorin, Kwara State, Nigeria; b Department of Physics, Covenant University, Ota, Ogun State, Nigeria

**Keywords:** Environmental radioactivity, Radioactivity, Radiological risk assessment, Granite mining field, Super-Spec RS-125, Construction purposes, North-Central Nigeria

## Abstract

In this study, a well calibrated Super-Spec (RS-125) gamma spectrometer was used to measure the activity concentrations of *^40^K, ^238^U, ^232^Th* and gamma doses rate at 1 m above the ground level over a granite mining field in Asa, Kwara State, North-central Nigeria. Measurements were carried out in 50 randomly selected sample points. The overall mean activity concentrations of *^40^K, ^238^U, ^232^Th* and gamma dose are 570.91, 42.86, 18.15 *Bqkg^−1^*, and 60.11 *nGyh^−1^* respectively. The results of the activity concentrations were used to estimate the corresponding radiation hazard parameters to assess the suitability of the granite for building and construction purposes. The data in this study could serve as the baseline radiological data of the region for future references.

• Activity concentrations of *^40^K,^238^U,^232^Th* and gamma doses were measured over a granite mining field in Asa.
• The total mean activity concentrations of the radioisotopes and gamma dose are 570.91, 42.86, 18.15 *Bqkg^−1^*, and 60.11 *nGyh^−1^* respectively.
• The radiological hazards are higher than the recommended permissible limits.

## Graphical abstract

**Figure:**
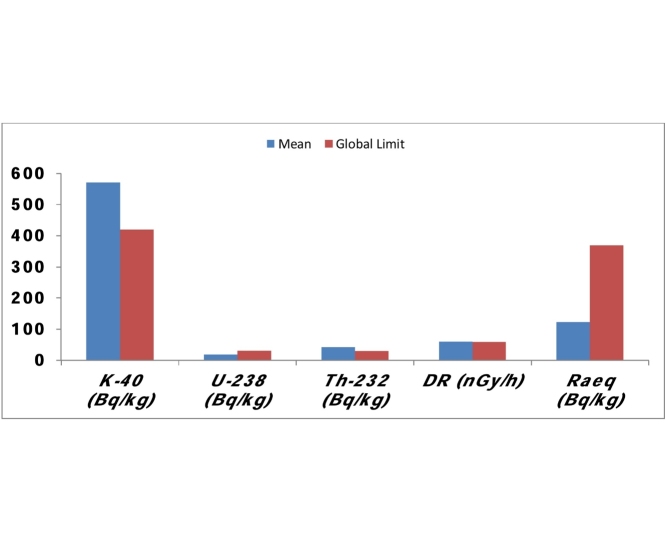

## Specification Table

**Table:** 

Subject Area:	*Environmental Sciences*
More specific subject area:	*Radiation and Health*
Method name:	*Environmental Radioactivity*
Name and reference of original method:	*Radiometrics*
Resource availability:	*Super-Spec Gamma RS-125*

## Method details

Natural radionuclides are broadly dispersed in the Earth crust. They are found in significant concentrations in many mineral rocks. Granites, just like other mineral rocks, may possibly hold deposits of natural radionuclides like ^238^U, ^232^Th, their progenies and the non-series ^40^K [1,2]. The activity concentrations of these radionuclides may differ even within a particular block of granite. If present, these radionuclides will decay to give off radon and some amounts of gamma and beta radiations. Human exposure to ionizing radiation resulting from these radionuclides and their progenies can cause cancer and other radiation health effects, damaging critical organs of the body which could even lead to death [1,[3], [4], [5]]. For granites used for building and construction of houses, these dangerous radiations will be released over the lifetime of using such buildings. So the knowledge of the concentrations of these radionuclides in building materials is fundamental for estimating the level of public exposure to radiations, since most residents spend approximately 80% of their time indoors. In order to reduce these radiation risks, the United State Environmental Protection Agency recommended that all houses should be tested for these radionuclides, whether they contains granite countertops or not [1]. Such an action is not economically feasible for a third world country like Nigeria. So researchers resolve to monitoring and assessments of the mine fields where the building materials (mineral rocks or soils) are mined originally and their finished products.

The levels of *^238^U, ^232^Th*, their respective progenies and the non-series *^40^K* have been studied in different building materials (both raw and finished products) from different parts of the country [[6], [7], [8], [9], [10], [11], [12], [13], [14], [15], [16], [17], [18], [19], [20], [21], [22]], but none has been carried out in Kwara State despite the increasing level of granite mining and usage in this part of the country. Also, data from University of Ilorin Teaching Hospital (UITH) shows that 74 different cancers of 2246 (891 male and 1355 female) cancer patients within the age of 1–105 were recorded at the University of Ilorin Teaching Hospital (UITH) cancer registry between the period of 2007 and 2016 [23]. Therefore, a pioneer study which is based on internationally verified methodology regarding assessment of radiological health implications on the general populace due to granite mining in this part of the country is apposite.

## Study area

**Fig. 1:**
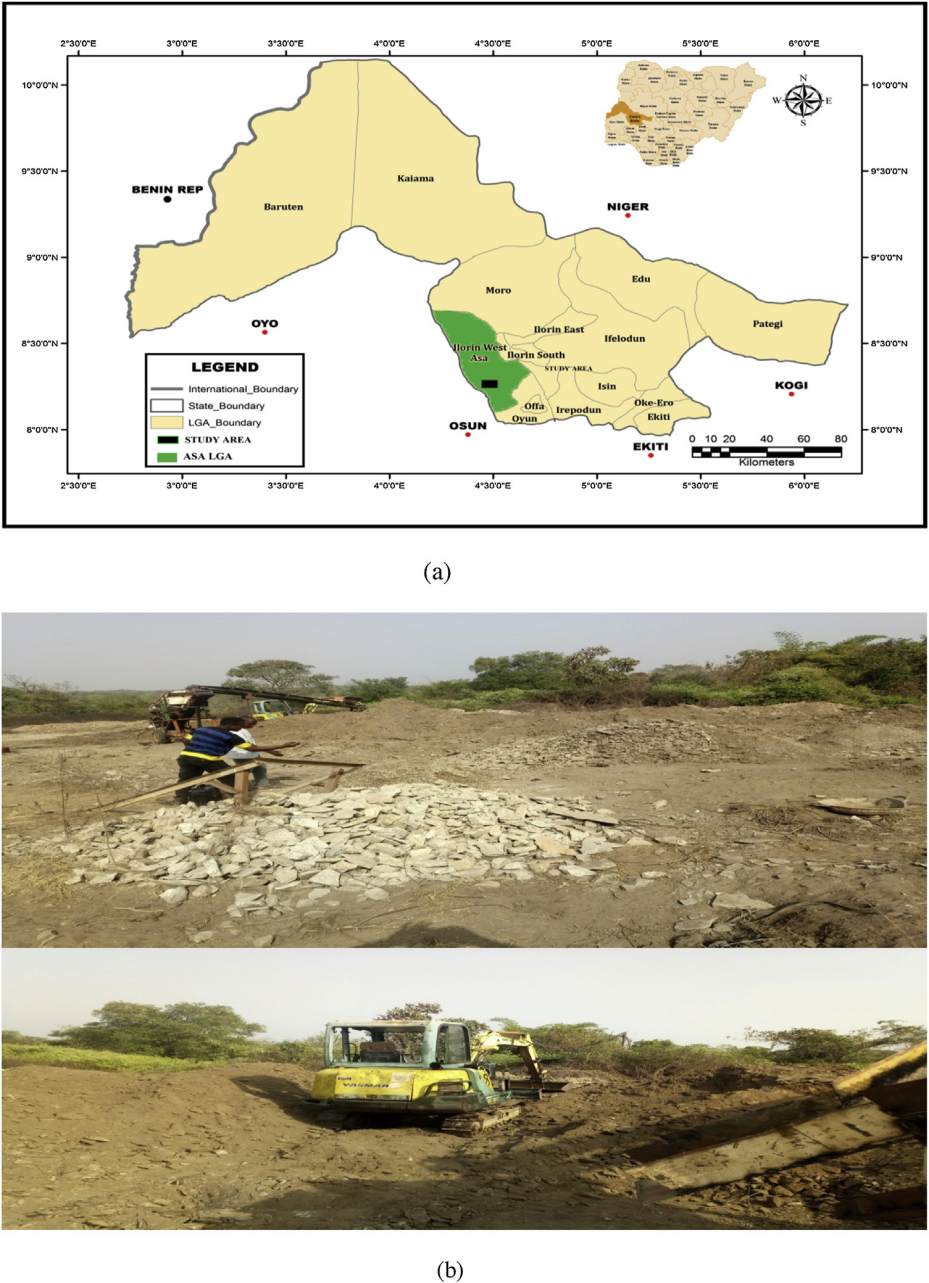
(a) Geological map of Nigeria showing the survey area (b) Granite mining field in Asa LGA, Kwara state, Nigeria.

Asa is a Local Government Area in Kwara State, Nigeria. It has an area of 1286 km² and a population of 126,435 according to 2006 census. It is located at the southwestern part of Kwara State and it is surrounded by Moro local government to the north, Oyun and Offa local government to the South and Ilorin west local government to the East. The study area lies between latitudes 4^0^12’N and 4^0^29’N and longitudes 8^0^7’E and 8^0^42’E (Fig. 1a and b). The study area is underlain by basement complex rock. The soils are formed from basement complex rocks (metamorphic and igneous rocks) which is about 95%. The metamorphic rocks consist of biotite gnesiss, banded gnesiss, quartzite augitegnesiss and granitic gnesiss. The intrusive rock comprises of pegmatite and vein quartz [[24], [25], [26]]. Detail geology of the study area can be found in [[24], [25], [26], [27], [28]].

## Materials and methods

### Field survey

For the in situ measurements of activity concentrations of ^40^K, ^232^Th, ^238^U and the radiation dose exposures, Super SPEC RS-125 spectrometer with large 2.0 × 2.0 NaI crystal was used. The measurement of the activity concentration of the radionuclides was carried out at about 1 m above the topsoil [15,29]. The RS-125 is a transportable handheld radiation detector with high accuracy and likely error of about 5%. It presents superior integrated design with big detector, good sensitivity and easy to use. The RS-125 is manufactured by Canadian Geophysical Institute and it comes with a large data storage which allows one to take multiple readings with ease. The RS-125 spectrometer was calibrated in accordance with Canadian Geophysical Institute i.e., the instrument was calibrated on 1 × 1 m test pads, which employs 5 min spectra accumulation on potassium, uranium and thorium pads and 10 min accumulation on the Background pad. It makes use of sodiumiodide (NaI) crystal doped with thallium [Tl] as activator. The energy range of the instrument, is from 30 to 3000 keV, which is enough to detect most of the radiation giving off from the terrestrial sources (i.e. *^214^Bi* (609.31 and 1764.49 *keV*) gamma rays to determine 238U, *^212^Pb* (238.63 *keV*), ^208^Tl (583.19 *keV*) and *^228^Ac* (911.21 *keV*) gamma rays to determine ^232^Th and the photopeaks of ^40^K which occours in the background spectrum at 1460.83 keV). The detection of gamma-ray from cosmic ray is small and negligible due to the detector’s low response to high-energy gamma radiation. The total count of 120 s per assay was employed for best accuracy as stated in Radiation Solutions Inc [15]. The assay mode of the instrument gives the activity concentration of *^40^K* in percentage (%), *^238^U* and *^232^Th* in part per million (ppm). The data was converted to the conventional unit *Bqkg^−1^* using conversion factors given by [15,30].

In this work, four (4) readings were recorded at each data point at the interval of 120 s. 50 sample points were recorded to cover the area of the mining field. The field was divided into grids of approximately equal size (i.e. 50 semi-rectangular boxes) with each box representing a data collection point. At each of these samples location (point), the coordinate and elevation were determined using a global positioning system (GPSMAP78). More details about the instrument can be found in [15,17,19,29].

### Estimation of the radiological impact parameters (RIP)

#### Radium equivalent activity index (Ra_eq_)

The distributions of the measured radionuclides are not uniform in the environment. So exposure to radiation has been defined in terms of radium equivalent activity (*Ra_eq_*) in *Bqkg^−1^* to compare the specific activity of materials containing different amounts of *^238^U, ^232^Th* and *^40^K*. This is based on the assumption that 1 *Bqkg^−1^* of *^238^U*, 0.7 *Bqkg^−1^* of *^232^Th* and 13 *Bqkg^−1^* of *^40^K* produce the same radiation dose rates. This allows a single number to be used to represent the gamma output due to different combination of *^238^U, ^232^Th* and *^40^K* in the granite material. The Ra*_eq_* was calculated using Eq. (1) [31,32]:

(1) $${Ra}_{eq}\;=\;C_{U}\;+\;1.43C_{Th}\;+\;0.077C_{K}$$

where *C_u_, C_Th_* and *C_K_* are the radioactivity concentration in *Bqkg^−1^* for *^238^U, ^232^Th* and *^40^K* respectively. The average value of 370 *Bqkg^-1^* is recommended normal background radiation value [31].

#### Radiation hazard indices

Eq. (2) and (3) were used to calculate the external radiation hazard (*H_ext_*) and the internal radiation hazard (*H_int_*).

(2) $$H_{ext}\;=\left(\frac{C_{U}}{370}\right)+\left(\frac{C_{Th}}{259}\right)+\left(\frac{C_{K}}{4810}\right)$$

(3) $$H_{int}\;=\left(\frac{C_{U}}{185}\right)+\left(\frac{C_{Th}}{259}\right)+\left(\frac{C_{K}}{4810}\right)$$

where *C_u_, C_Th_* and *C_K_* are as defined in Eq. (1) above.

For the radiation hazard to be small, both *H_int_* and *H_ext_* ought to be less than 1. Natural radioactive elements in soil generates external field to which the general populace are exposed. *H_ext_* equal to unity translates to the upper limit of radium equivalent dose (370 *Bqkg^−1^*) [19,31,32].

#### Absorbed dose rate

At 1 m height above the ground level, it is assumed that the naturally occurring radionuclides will have a uniform distribution. The outdoor absorbed dose rate at 1 m above the ground is calculated using Eq. (4) [2,15,31].

(4) $$D_{outdoor}(nGyh^{-1})\;=\;{0.462C}_{u}+{0.604C}_{Th}+{0.041C}_{K}$$

But fortunately, this outdoor dose rate was measured in situ using the RS-125 Gamma Spec.

The granite from Asa LGA as highlighted earlier, is primarily used for building purposes. As a result, the indoor radiation dose rate in a typical building of 4 × 5 × 2.8 m room size, with wall thickness of about 20 cm was calculated using Eq. (5) [13]:

(5) $$D_{indoor}(nGyh^{-1})\;=\;{0.92C}_{u}+{1.1C}_{Th}+0.08C_{K}$$

where *C_u_, C_Th_* and *C_K_* are as defined earlier.

#### Annual effective dose (AED)

The annual effective dose received indoor and outdoor by a member of the public was calculated from dose rates given in Eqs. (6) and (7). Dose conversion factor of 0.7 *Sv Gy^−1^* and occupancy factor for outdoor and indoor as 0.2 and 0.8 were adopted [13,31].

(6) AED_outdoor_ (mSvy^−1^) = D_outdoor_ (nGy h^−1^) × 8760 h × 0.7 (Sv Gy^−1^)×0.2 × 10^-6^

(7) AED_intdoor_ (mSvy^−1^) = D_indoor_ (nGy h^−1^) × 8760 h × 0.7 (Sv Gy^−1^)×0.8 × 10^-6^

#### Excess Lifetime Cancer Risk (ELCR)

The Excess Lifetime Cancer Risk (*ELCR*) was calculated using Eq. (8):

(8) *ELCR = AED_indoor_ ×DL × RF*

where, *AED_indoor_* is the indoor Annual Equivalent Dose, *DL* is the average duration of life (estimated to 70 years) and *RF* is the Risk Factor ( *Sv^−1^*), i.e. fatal cancer risk per Sievert. ICRP uses *RF* as 0.05 for stochastic effects for the general public [19,31,32].

#### Annual gonadal equivalent dose (AGED)

An increase in *AGED* has been known to result in leukemia which is very fatal. This hazard parameter for the residents using the granite for building was evaluated using Eq. (9) [19,31,32]:

(9) *AGED* (*μSvy^−1^*) = 3.09*C_U_* + 4.18*C_Th_* + 0.314*C_K_*

where *C_U_, C_Th_,* and *C_K_* maintain their usual definitions.

#### Representative level index (RLI)

*RLI* value of 1 corresponds to an *AED* of less than or equal to 1 *mSv*. Thus, *RLI* is a radiological impact parameter for screening materials used for building construction and the *RLI* was estimated using Eq. 10 [31,32].

(10) $$RLI=\;\frac{C_{u}}{150}+\frac{C_{Th}}{100}+\frac{C_{k}}{1500}\leq 1$$

where *C_U_, C_Th_,* and *C_K_* maintain their usual definition.

## Method descriptions

**Fig. 2:**
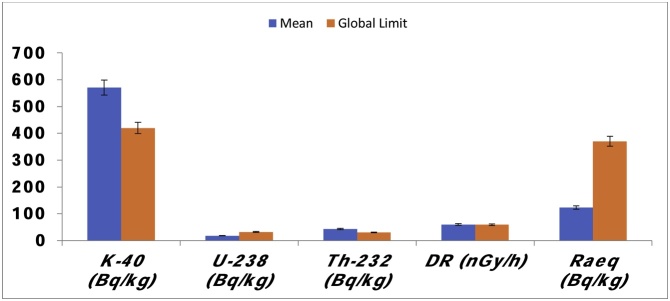
Mean activity concentrations of *^40^K, ^232^Th & ^238^U,* Dose rate (*DR*) and the Radium equivalent.

The record of the measured activity concentrations of *^40^K, ^238^U* and *^232^Th*, the gamma dose rate, the elevations and the estimated radium equivalent activity index for the 50 sample locations is presented in Table 1. The mean activity concentration of *^40^K* was observed to dominate the *^238^U* and *^232^Th* mean activities. The activity concentration of ^40^K ranges between 250.40 ± 6.0 and 970.30 ± 6.0 *Bqkg^−1^* with an average value of 570.91 *Bqkg^−1^*. For *^238^U*, the measured activities range between 1.24 ± 0.1 and 61.75 ± 3.4 with mean value of 18.15, while for *^232^Th* it ranges between 8.53 ± 0.5 and 76.33 ± 5.2 with an average value of 42.86 *Bqkg^−1^*. The estimated mean value for *^40^K* was relatively higher than the global average of 420.00 *Bqkg^−1^* for normal background radiation levels recommended by [31] as shown in Fig. 2. It was observed that the measured activity concentration of *^40^K* were lower than the global limit in just 8 (16%) locations out of the 50. Surprisingly, all the measured and the mean activity concentrations of *^238^U* are lower than the global average of 32.00 *Bqkg^−1^* [31]. However, the mean activity concentration of *^232^Th* was found to higher than the given global average of 30.00 *Bqkg^−1^*. As a matter of fact, the measured values of the activity concentrations are higher than the recommended limit in about 80% (40 out 50) of the sample points. This is a reason for concern because considerable enrichment or increase in the concentration of *^232^Th* will enhance the level of the background radiation and maybe render the mineral rock unfit for use in building and construction purposes. The maximum, minimum and the average value for the measured outdoor dose rate are 85.30 ± 2.0, 40.10 ± 0.1 and 60.11 *nGyhr^−1^* respectively. This mean value for the outdoor dose is higher than the recommended permissible value of 59 *nGyh^−1^* recommended [31]. Fig. 2 revealed that the granite mine field is enriched with potassium and thorium which causes the gamma dose rate to be high. This high background ionizing radiation has been reported to cause various kinds of cancers and cruel health related harms which may possibly lead to death [5,13,15,19].

**Table 1:** Measured activity concentrations of ^4^*^0^K, ^238^U, ^232^Th*, the absorbed dose rates (*DR*) and the Radium equivalent activity from Asa LGA.

SAMPLE CODE	Latitude *⁰N*	Longitude *⁰E*	Elvtn *(m)*	*DR* *(nGyh^−1^)*	*^40^K* *(Bqkg^−1^)*	*^238^U (Bqkg^−1^)*	*^232^Th (Bqkg^−1^)*	*Ra_eq_ (Bqkg^−1^)*
ASAS1	8⁰21.296'	4⁰24.023'	359	55.70 ± 0.4	500.80 ± 7.0	25.94 ± 1.0	35.32 ± 2.0	115.01
ASAS2	8⁰21.297'	4⁰24.026'	358	59.60 ± 3.2	532.10 ± 5.0	11.12 ± 0.1	48.72 ± 2.4	121.76
ASAS3	8⁰21.297'	4⁰24.028'	358	59.70 ± 2.0	626.00 ± 6.0	22.23 ± 1.0	36.95 ± 3.0	123.26
ASAS4	8⁰21.298'	4⁰24.031'	359	78.70 ± 5.0	688.60 ± 3.0	39.52 ± 1.2	48.31 ± 3.0	161.63
ASAS5	8⁰21.298'	4⁰24.032'	360	65.10 ± 2.1	657.30 ± 9.0	39.52 ± 2.0	30.04 ± 2.0	133.10
ASAS6	8⁰21.298'	4⁰24.033'	360	52.30 ± 2.0	532.10 ± 7.0	24.70 ± 2.1	29.23 ± 1.0	107.47
ASAS7	8⁰21.298'	4⁰24.035'	360	60.70 ± 1.0	657.30 ± 6.0	25.94 ± 1.0	32.89 ± 1.0	123.57
ASAS8	8⁰21.299'	4⁰24.037'	360	49.10 ± 3.0	532.10 ± 8.0	1.24 ± 1.0	41.41 ± 2.0	101.43
ASAS9	8⁰21.299'	4⁰24.037'	359	53.50 ± 2.0	438.20 ± 6.0	18.53 ± 2.1	40.60 ± 2.0	110.32
ASAS10	8⁰21.299'	4⁰24.040'	360	45.20 ± 1.0	438.20 ± 7.0	1.24 ± 1.0	41.01 ± 2.0	93.61
ASAS11	8⁰21.298'	4⁰24.042'	361	49.60 ± 1.0	532.10 ± 4.0	30.88 ± 2.1	21.11 ± 1.0	102.04
ASAS12	8⁰21.297'	4⁰24.040'	361	58.00 ± 1.0	688.60 ± 6.0	8.65 ± 1.0	38.57 ± 2.0	116.82
ASAS13	8⁰21.297'	4⁰24.038'	361	60.40 ± 1.0	406.90 ± 7.0	25.94 ± 1.0	48.31 ± 3.0	126.36
ASAS14	8⁰21.296'	4⁰24.036'	362	41.90 ± 1.0	438.20 ± 7.0	2.47 ± 1.0	33.29 ± 2.0	83.82
ASAS15	8⁰21.296'	4⁰24.034'	360	51.40 ± 1.0	438.20 ± 4.0	18.53 ± 1.1	38.16 ± 2.0	106.84
ASAS16	8⁰21.295'	4⁰24.033'	359	77.70 ± 1.2	657.30 ± 5.0	1.24 ± 1.0	76.33 ± 5.2	161.00
ASAS17	8⁰21.295'	4⁰24.031'	359	63.70 ± 1.4	719.90 ± 5.0	6.18 ± 1.0	46.69 ± 1.2	128.37
ASAS18	8⁰21.294'	4⁰24.031'	360	60.70 ± 2.0	688.60 ± 5.0	17.29 ± 1.2	36.95 ± 2.0	123.14
ASAS19	8⁰21.294'	4⁰24.030'	359	74.60 ± 2.0	688.60 ± 4.0	1.24 ± 0.1	68.61 ± 3.2	152.38
ASAS20	8⁰21.293'	4⁰24.028'	359	49.50 ± 4.0	438.20 ± 3.0	13.59 ± 1.0	38.16 ± 2.0	101.90
ASAS21	8⁰21.291'	4⁰24.028'	360	40.10 ± 0.1	250.40 ± 6.0	23.47 ± 2.1	28.83 ± 2.1	83.97
ASAS22	8⁰21.291'	4⁰24.030'	359	63.50 ± 6.0	626.00 ± 7.0	39.52 ± 2.4	29.64 ± 1.0	130.10
ASAS23	8⁰21.291'	4⁰24.030'	359	55.50 ± 2.0	594.70 ± 7.0	1.24 ± 1.0	45.47 ± 2.0	112.05
ASAS24	8⁰21.292'	4⁰24.033'	358	61.90 ± 2.0	626.00 ± 6.0	1.24 ± 1.0	53.19 ± 3.0	125.49
ASAS25	8⁰21.292'	4⁰24.034'	359	51.40 ± 2.5	313.00 ± 4.0	23.47 ± 2.0	41.82 ± 2.4	107.37
ASAS26	8⁰21.293'	4⁰24.035'	356	56.10 ± 2.3	532.10 ± 7.0	9.88 ± 1.0	44.66 ± 1.0	114.72
ASAS27	8⁰21.293'	4⁰24.036'	358	52.10 ± 2.4	281.70 ± 5.0	43.23 ± 2.0	32.07 ± 1.3	110.78
ASAS28	8⁰21.293'	4⁰24.040'	358	54.60 ± 2.1	406.90 ± 5.0	35.82 ± 2.0	33.70 ± 1.0	115.33
ASAS29	8⁰21.295'	4⁰24.042'	360	55.60 ± 5.0	532.10 ± 7.0	29.64 ± 1.0	30.04 ± 2.0	113.57
ASAS30	8⁰21.296'	4⁰24.043'	359	46.50 ± 2.0	344.30 ± 7.0	1.24 ± 1.0	49.53 ± 2.0	98.58
ASAS31	8⁰21.308'	4⁰24.039'	354	55.10 ± 2.0	626.00 ± 5.0	1.24 ± 1.0	43.04 ± 2.0	110.98
ASAS32	8⁰21.307'	4⁰24.037'	356	58.80 ± 2.0	782.50 ± 5.0	1.24 ± 0.1	38.98 ± 1.1	117.22
ASAS33	8⁰21.307'	4⁰24.037'	356	46.00 ± 2.0	406.90 ± 6.0	8.65 ± 2.0	38.16 ± 1.0	94.55
ASAS34	8⁰21.306'	4⁰24.035'	355	49.60 ± 1.0	406.90 ± 7.0	61.75 ± 3.4	8.53 ± 0.5	105.27
ASAS35	8⁰21.306'	4⁰24.032'	355	85.30 ± 2.0	751.20 ± 7.0	34.58 ± 1.0	58.06 ± 5.2	175.45
ASAS36	8⁰21.304'	4⁰24.030'	356	81.30 ± 4.0	657.30 ± 7.0	54.34 ± 2.0	45.47 ± 3.0	169.98
ASAS37	8⁰21.304'	4⁰24.030'	356	85.20 ± 6.0	970.30 ± 6.0	1.24 ± 1.0	66.99 ± 2.0	171.74
ASAS38	8⁰21.303'	4⁰24.028'	357	55.70 ± 2.0	657.30 ± 6.0	19.76 ± 1.0	29.23 ± 1.0	112.17
ASAS39	8⁰21.303'	4⁰24.024'	358	49.10 ± 2.0	594.70 ± 2.0	22.23 ± 1.3	22.33 ± 1.2	99.95
ASAS40	8⁰21.303'	4⁰24.023'	358	55.90 ± 1.0	563.40 ± 7.0	35.82 ± 1.2	23.95 ± 1.0	113.45
ASAS41	8⁰21.304'	4⁰24.023'	359	69.80 ± 1.0	657.30 ± 4.0	1.24 ± 1.0	62.93 ± 3.1	141.84
ASAS42	8⁰21.304'	4⁰24.024'	357	70.10 ± 4.0	500.80 ± 4.0	12.35 ± 1.3	66.18 ± 3.0	145.55
ASAS43	8⁰21.306'	4⁰24.025'	357	63.80 ± 2.0	657.30 ± 5.0	7.41 ± 1.1	52.37 ± 4.2	132.92
ASAS44	8⁰21.307'	4⁰24.027'	358	67.20 ± 2.0	688.60 ± 5.0	1.24 ± 1.0	56.84 ± 2.0	135.54
ASAS45	8⁰21.308'	4⁰24.029'	358	73.50 ± 4.0	782.50 ± 6.0	29.64 ± 1.0	42.22 ± 2.3	150.27
ASAS46	8⁰21.309'	4⁰24.031'	359	74.00 ± 3.0	688.60 ± 3.0	30.88 ± 2.2	48.31 ± 1.0	152.99
ASAS47	8⁰21.309'	4⁰24.032'	358	67.20 ± 2.0	688.60 ± 2.0	8.65 ± 1.0	55.62 ± 2.0	141.21
ASAS48	8⁰21.311'	4⁰24.036'	358	70.60 ± 2.0	657.30 ± 5.0	1.24 ± 1.0	61.71 ± 4.1	140.10
ASAS49	8⁰21.310'	4⁰24.039'	358	52.70 ± 1.0	563.40 ± 4.0	4.94 ± 1.0	41.01 ± 2.0	106.96
ASAS50	8⁰21.312'	4⁰24.043'	361	70.00 ± 1.0	438.20 ± 5.0	24.70 ± 1.0	61.31 ± 3.1	146.11
**Min**			**354**	**40.10 ± 0.1**	**250.40 ± 6.0**	**1.24 ± 0.1**	**8.53 ± 0.5**	**83.82**
**Max**			**362**	**85.30 ± 2.0**	**970.30 ± 6.0**	**61.75 ± 3.4**	**76.33 ± 5.2**	**175.45**
**Mean**			**359**	**60.11**	**570.91**	**18.15**	**42.86**	**123.40**
**GLOBAL AVERAGE** [31]			–	**59.00**	**420.00**	**32.00**	**30.00**	**370.00**

We conducted a correlation analysis to study the relationship between these measured radionuclides and the gamma dose rate. The result of the correlation analysis which is presented in Table 2, were classified according to the correlation coefficient R [33]. A significant correlation was found to exist between *DR* and *^40^K* (R = 0.7259), *DR* and *^232^Th* (R = 0.6768) and *^232^Th* and *^238^U* (0.5450). While weak correlation was observed between *^40^K* and *^232^Th* (R = 0.3768) and insignificant correlation was observed to exist between others. The correlation results confirm that the granite mine field is endowed with potassium and thorium, and they contributed significantly to the gamma dose received from the field than *^238^U*. However, the significant correlation observed between *^232^Th* and *^238^U* could mean that they share common origin during the rock formation.

**Table 2:** Pearson’s correlation matrix showing the relationship between the measured radionuclides and the gamma dose rate at the granite mine field.

	*DR (nGyh^−1^)*	*^40^K (Bqkg^−1^)*	*^238^U (Bqkg^−1^)*	*^232^Th (Bqkg^−1^)*
*DR (nGyh^−1^)*	1.0000			
*^40^K (Bqkg^−1^)*	0.7259	1.0000		
*^238^U (Bqkg^−1^)*	0.0775	0.1975	1.0000	
*^232^Th (Bqkg^−1^)*	0.6768	0.3768	0.5450	1.0000

The results of the estimated radiological parameters R*a_eq_, H_int_, H_ext_ D_in_, D_out_, AED_indoor_, AED_outdoor_, ELCR, AGED* and *RLI* respectively are presented in Table 3. The estimated values for the radium equivalent (*Ra_eq_*) ranges between 175.45 and 83.82 *Bqkg^−1^* with an average value of 123.40 *Bqkg^−1^*. The average value of *Ra_eq_* is lower than the limit of 370 *Bqkg^−1^* recommended by UNSCEAR [31] for materials considered safe for the construction of buildings. The calculated highest, lowest and mean values of the external radiation hazard (*H_ext_*) and the internal radiation hazard (*H_int_*) are below unity. The mean values for the *D_out_* and *EAD_outdoor_* are 57.68 *nGyh^−1^* and 0.07 *mSvy^−1^* respectively. These values are about the recommended values of 59.00 *nGyh^−1^* and 0.07 *mSvy^−1^* for *D_out_* and *EAD_outdoor_* respectively. The indoor gamma dose (*D_in_*) received by the general populace due to the radionuclides concentration in the granite ranges between 155.77 and 73.33 *nGyh^−1^* with mean value of 109.52 *nGyh^−1^*. The estimated mean value of *EAD_indoo_*_r_ was found to be 0.54 *mSvy^−1^*. These mean values of *D_in_* and *EAD_indoor_* are well above the limits of 84.00 *nGyh^−1^* and 0.41 *mSvy^−1^* respectively [2,13,15,19,31]. This reveals that there is danger of indoor gamma radiation exposure is much and the general public is not safe from overexposure to indoor ionizing radiation if the granite is used for building purposes.

**Table 3:** Summary of the estimated radiological parameters (RIP).

SAMPLE CODE	*D_in_ (nGyh^−1^)*	*D_out_ (nGyh^−1^)*	*AED_outdoor_* *(mSvy^−1^)*	*AED_indoor_* *(mSvy^−1^)*	*H_ext_*	*H_int_*	*RLI*	*ELCR* *(X 10^−3^)*	*AGED (mSvy^−1^)*
ASAS1	102.78	53.85	0.07	0.50	0.31	0.38	0.86	1.76	0.39
ASAS2	106.39	56.38	0.07	0.52	0.33	0.36	0.92	1.83	0.41
ASAS3	111.17	58.25	0.07	0.55	0.34	0.40	0.94	1.91	0.42
ASAS4	144.59	75.67	0.09	0.71	0.44	0.55	1.21	2.48	0.54
ASAS5	121.99	63.35	0.08	0.60	0.36	0.47	1.01	2.09	0.45
ASAS6	97.45	50.88	0.06	0.48	0.29	0.36	0.81	1.67	0.37
ASAS7	112.62	58.79	0.07	0.55	0.34	0.41	0.94	1.93	0.42
ASAS8	89.26	47.40	0.06	0.44	0.28	0.28	0.78	1.53	0.34
ASAS9	96.76	51.05	0.06	0.47	0.30	0.35	0.82	1.66	0.36
ASAS10	81.30	43.30	0.05	0.40	0.26	0.26	0.71	1.40	0.31
ASAS11	94.20	48.83	0.06	0.46	0.28	0.36	0.77	1.62	0.35
ASAS12	105.47	55.52	0.07	0.52	0.32	0.34	0.90	1.81	0.40
ASAS13	109.56	57.85	0.07	0.54	0.34	0.41	0.93	1.88	0.41
ASAS14	73.95	39.22	0.05	0.36	0.23	0.24	0.64	1.27	0.28
ASAS15	94.08	49.58	0.06	0.46	0.29	0.34	0.80	1.62	0.35
ASAS16	137.68	73.62	0.09	0.68	0.44	0.44	1.21	2.36	0.53
ASAS17	114.63	60.57	0.07	0.56	0.35	0.37	0.99	1.97	0.44
ASAS18	111.64	58.54	0.07	0.55	0.34	0.38	0.95	1.92	0.42
ASAS19	131.70	70.25	0.09	0.65	0.42	0.42	1.16	2.26	0.51
ASAS20	89.53	47.29	0.06	0.44	0.28	0.31	0.77	1.54	0.34
ASAS21	73.33	38.52	0.05	0.36	0.23	0.29	0.61	1.26	0.27
ASAS22	119.04	61.83	0.08	0.58	0.35	0.46	0.98	2.04	0.44
ASAS23	98.73	52.42	0.06	0.48	0.31	0.31	0.86	1.70	0.38
ASAS24	109.72	58.36	0.07	0.54	0.34	0.35	0.96	1.88	0.42
ASAS25	92.63	48.93	0.06	0.45	0.29	0.36	0.79	1.59	0.35
ASAS26	100.78	53.36	0.07	0.49	0.31	0.34	0.87	1.73	0.38
ASAS27	97.58	50.89	0.06	0.48	0.30	0.42	0.80	1.68	0.36
ASAS28	102.57	53.58	0.07	0.50	0.31	0.41	0.85	1.76	0.38
ASAS29	102.89	53.66	0.07	0.50	0.31	0.39	0.86	1.77	0.38
ASAS30	83.17	44.60	0.05	0.41	0.27	0.27	0.73	1.43	0.32
ASAS31	98.56	52.23	0.06	0.48	0.30	0.31	0.86	1.69	0.38
ASAS32	106.61	56.19	0.07	0.52	0.32	0.32	0.92	1.83	0.41
ASAS33	82.49	43.73	0.05	0.40	0.26	0.28	0.71	1.42	0.31
ASAS34	98.74	50.36	0.06	0.48	0.29	0.45	0.77	1.70	0.35
ASAS35	155.77	81.84	0.10	0.76	0.48	0.57	1.32	2.67	0.59
ASAS36	152.60	79.52	0.10	0.75	0.46	0.61	1.26	2.62	0.56
ASAS37	152.45	80.81	0.10	0.75	0.47	0.47	1.33	2.62	0.59
ASAS38	102.92	53.73	0.07	0.50	0.31	0.36	0.87	1.77	0.39
ASAS39	92.59	48.14	0.06	0.45	0.27	0.33	0.77	1.59	0.35
ASAS40	104.37	54.11	0.07	0.51	0.31	0.41	0.86	1.79	0.39
ASAS41	122.94	65.53	0.08	0.60	0.39	0.39	1.08	2.11	0.47
ASAS42	124.22	66.21	0.08	0.61	0.40	0.43	1.08	2.13	0.47
ASAS43	117.01	62.01	0.08	0.57	0.36	0.38	1.01	2.01	0.45
ASAS44	118.75	63.13	0.08	0.58	0.37	0.37	1.04	2.04	0.46
ASAS45	136.32	71.28	0.09	0.67	0.41	0.49	1.15	2.34	0.51
ASAS46	136.64	71.68	0.09	0.67	0.42	0.50	1.15	2.35	0.51
ASAS47	124.23	65.82	0.08	0.61	0.38	0.41	1.08	2.13	0.48
ASAS48	121.60	64.79	0.08	0.60	0.38	0.39	1.07	2.09	0.47
ASAS49	94.72	50.15	0.06	0.46	0.29	0.30	0.82	1.63	0.36
ASAS50	125.22	66.41	0.08	0.61	0.40	0.46	1.07	2.15	0.47
**Min**	73.33	38.52	0.05	0.36	0.23	0.24	0.61	1.26	0.27
**Max**	155.77	81.84	0.10	0.76	0.48	0.61	1.33	2.67	0.59
**Mean**	**109.52**	**57.68**	**0.07**	**0.54**	**0.34**	**0.39**	**0.93**	**1.88**	**0.41**
**WORLD LIMIT** [31]	**84.00**	**59.00**	**0.07**	**0.41**	**≤1**	**≤1**	**≤1**	**3.75**	**0.30**

**Fig. 3:**
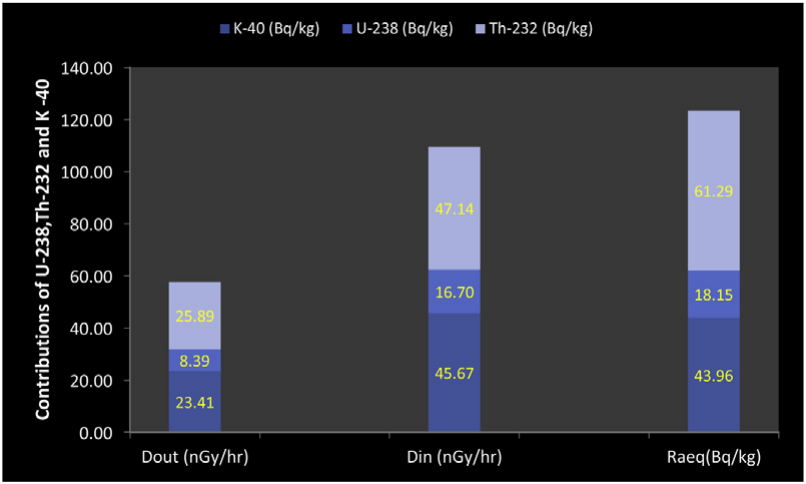
Contributions of ^40^K, ^238^U and ^232^Th to D_out_, D_in_ and Ra_eq_.

**Fig. 4:**
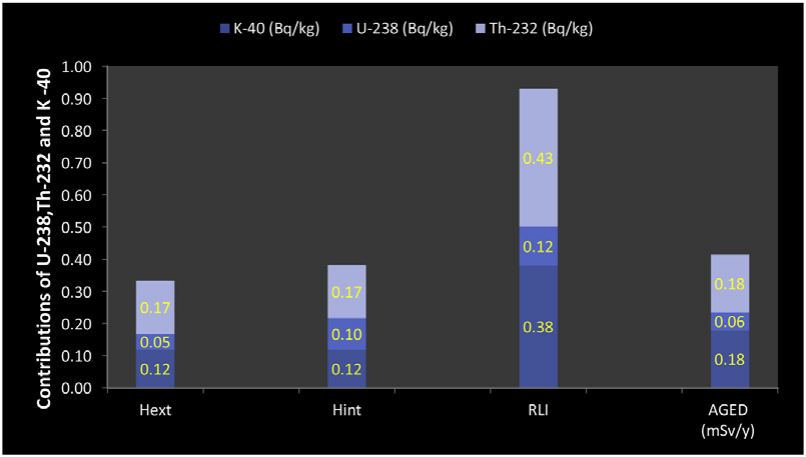
Contributions of *^40^K, ^238^U and ^232^Th* to *H_ext_, H_in_, RLI* and *AGED.*

The mean value for the Excess Lifetime Cancer Risk (ELCR) was estimated and found to be below the recommended limits of 3.75 × 10^−3^. The maximum, minimum and mean values of the *AGED* for the residents using the granite for building are 0.59, 0.27 and 0.41 *mSvy^-1^* respectively. The mean value of the *AGED* is higher than the recommended limit of 0.32 *mSvy^-1^*. This high value of *AGED* further augmented our worry over the use of the granite from the mine field in Asa LGA for building purposes. The estimated *RLI* ranges between 1.33 and 0.61 with a mean value of 0.93. The estimated mean value is close to unity, so care should be taken in the use of the granite from this mine field for building. The contributions of *^40^K, ^238^U* and *^232^Th* to the *Ra_eq_, D_out_, D_in_, H_in_, H_ext_, RLI* and *AGED* were investigated and presented in Fig. 3, Fig. 4. It reveals that *^40^K* and *^234^Th* were the chief contributors to the radiological hazards.

## Conclusion

A well calibrated Super-Spec (RS-125) gamma spec was used to measure the activity concentrations of ^40^K, ^238^U, ^232^Th and gamma doses rate over a granite mining field in Asa, Kwara State, North-central Nigeria. The results of the activity concentrations were used to estimate the corresponding radiation hazard parameters to assess the suitability of the granite for building and construction purpose. The results of the activity concentrations showed that the mine field is loaded with thorium and potassium which as a result enhances the outdoor gamma radiation dose rate. The estimated mean values of *D_in_, EAD_indoor_* and *AGED* are above the recommended limits which follows that the danger of indoor gamma radiation exposure is high and the residents may not be safe from indoor ionizing radiation overexposure if the granite is used for building. Other hazard parameters are close to the recommended limits. The study therefore concludes that Nigerian Environmental Protection Agency (NEPA) and other regulatory bodies should implement specific statutory requirements and laws to regulate the high rate of mining activities in the State and the country at large. And in accordance with international recommendations quoted in the Basic Safety Series No.115 from the IAEA, the use of building materials containing enhanced concentrations of NORM should be controlled and restricted under the application of the radiation safety standards.

## Declaration of Competing Interest

The authors declare that they have no known competing financial interests or personal relationship that could have appeared to influence the work reported in this paper.
